# Healthcare use of young Finnish adults with mental disorders – profiles and profile membership determinants

**DOI:** 10.1186/s12875-024-02497-z

**Published:** 2024-07-04

**Authors:** Visa Väisänen, Outi Linnaranta, Timo Sinervo, Laura Hietapakka, Marko Elovainio

**Affiliations:** 1 Finnish Institute for Health and Welfare, Welfare State Research and Reform unit, Health and Social Service System Research team, Mannerheimintie 166, Helsinki, 00300 Finland; 2 Faculty of Social Sciences and Business Studies, Department of Health and Social Management, University of Eastern Finland, Yliopistonranta 8 E, Kuopio, 70210 Finland; 3 Finnish Institute for Health and Welfare, Equality unit, Mental health team, Mannerheimintie 166, Helsinki, 00300 Finland; 4 Faculty of Medicine, Research Program Unit, Department of Psychology, University of Helsinki, Haartmaninkatu 3, Helsinki, 00014 Finland

**Keywords:** Health care utilization, Young adult, Mental health disorder, Register study

## Abstract

**Background:**

Comprehensive, timely, and integrated primary care services have been proposed as a response to the increased demand for mental health and substance use services especially among young people. However, little is known about the care utilization patterns of young people with mental and substance use disorders. Our aim was to characterize profiles of care use in young Finnish adults with mental or substance use disorders, and the potential factors associated with the service use profiles.

**Methods:**

Primary and specialized care visits of young adults (16–29 years) diagnosed with a psychiatric or a substance use disorder (*n* = 7714) were retrieved from the national health care register from years 2020 and 2021. K-Means clustering was used to detect different profiles based on the utilization of care services. Multinomial logistic regression was used to analyze the factors associated with different profiles of care use.

**Results:**

Five different profiles were identified: low care use (75%), and use of principally primary health care (11%), student health services (9%), psychiatric services (5%), or substance use services (1%). Female gender was associated with membership in the primary health care focused profiles (OR 2.58 and OR 1.99), and patients in the primary health care and student health services profiles were associated with a better continuity of care (OR 1.04 and OR 1.05). Substance use disorders were associated with psychiatric service use (OR: 2.51) and substance use services (OR: 58.91). Living in smaller municipalities was associated with lower service use when comparing to the largest city.

**Conclusions:**

Young adults diagnosed with a psychiatric or a substance use disorder had remarkably different and heterogeneous care patterns. Most of the participants had low care utilization, indicating potential gaps in service use and care needs. Measures should be taken to ensure equal access to and availability of mental health services. The profiles that utilized the most services highlights the importance of integrated services and patient-oriented improvement of treatment.

## Background

Young adults report growing subjective anxiety and depression across Europe and worldwide [1, 2]. During the COVID-19 pandemic, a significant increase in anxiety and depressive symptoms as well as help seeking was detected among young adults [3, 4], and unfortunately, no plateau has been seen after the pandemic. In addition, increased knowledge about climate change, catastrophes such as earthquakes and large fires, and global increases in tensions have negatively affected the mental health of young adults [5, 6], who might hold a more negative outlook on the future.

Problems with mental health in adolescence commonly endanger social and professional development [7]. This, in turn, can result in significant individual and societal costs, including lost economic productivity, and an increased burden on health, education, social protection, and justice systems [8, 9]. Severe or chronic physical illness in adolescence is associated with mental illness and suicidality in adulthood [10]. Furthermore, mental health problems in youth can increase the likelihood of psychiatric illness and disability in adulthood, with approximately half of adult mental health problems originating in childhood and adolescence [11, 12]. In a previous Finnish study, having mental health problems in adolescence considerably increased costs due to services for both mental and physical health at 29 to 33 years [13].

In order to improve population health and reduce strain on our health systems, effective prevention and timely treatment of mental health and substance use disorders are needed [2, 14]. Many strategies and initiatives for mental health have been proposed and enacted. The focus has been on promoting person-centered mental health care [15] and benchmarking current systems [16]. In the latest WHO report [2], calls have been made to further integrate mental health into primary health care. The provision of comprehensive and multidisciplinary primary health care (PHC) with high continuity and integration of care has been identified as a pivotal component of quality care for mental health clients [17, 18].

In Finland, outpatient mental health services are provided in primary care health centers or as a separate unit outside health centers. In addition, social services, student health system, occupational care services, non-governmental organizations, and private service providers offer mental health services. Substance use services are concentrated in health centers, social services, and in an NGO, the A-Clinic Foundation [19]. The Finnish National Mental Health Strategy and Programme for Suicide Prevention for years 2020–2030 aims to strengthen the mental health of young people and offer low-threshold, evidence-based services [20, 21]. Recent developments in service provision have included the use of multidisciplinary mental health and substance use care teams [22] and low-threshold services [23] in primary care. Most adolescents visit only primary care, and the number of visits has increased even before the pandemic [24].

However, little is known about the actual care utilization of young people with mental and substance disorders, and about which patterns of care use (profiles) emerge in this population. Care utilization-based analysis can inform health service planning and care delivery and help understand the priorities of different population groups [25]. In addition, more information is needed on the provided care services and how different individual characteristics affect the use of both primary and specialized care. The findings can be used to deliver targeted programs to specific patient-groups, and ultimately improve care integration and reduce the fragmentation of the healthcare system [25, 26].

Two research questions were formulated:

- How is care service utilization segmented among young Finnish adults with mental health or substance use disorders?
- Which individual characteristics and psychiatric diagnoses are associated with different service use patterns?

## Methods

### Study setting

The study setting was a region in southern Finland with 200 000 inhabitants, ten municipalities, and one distinct large city with a central hospital, being a fairly generic region with a smaller area and larger population. Health and social services were arranged under a voluntary joint authority (except in some municipalities). During the data collection primary care was provided by private organizations for majority of the region. From primary services, referrals can be given to specialized care in a hospital or to other primary care or social services. Acute care is provided in both PHC (consultation without appointment in care centers during limited office hours) and specialized care (around-the-clock emergency departments in hospitals). Both health and social services, and PHC and specialized care, are organizationally well integrated in Finland [27]. However, the service system is also fragmented, especially in terms of funding sources and different service providers. There are multiple overlapping primary care providers: health centers operated by municipalities or joint authorities (wellbeing services counties since 2023), occupational health care, student health services, and private for-profit care [19]. The extensive role of occupational health care is especially unique, as it provides curative ambulatory primary care services for majority of the employed, being often both faster and cheaper than publicly provided care [28].

### Data

**Fig. 1:**
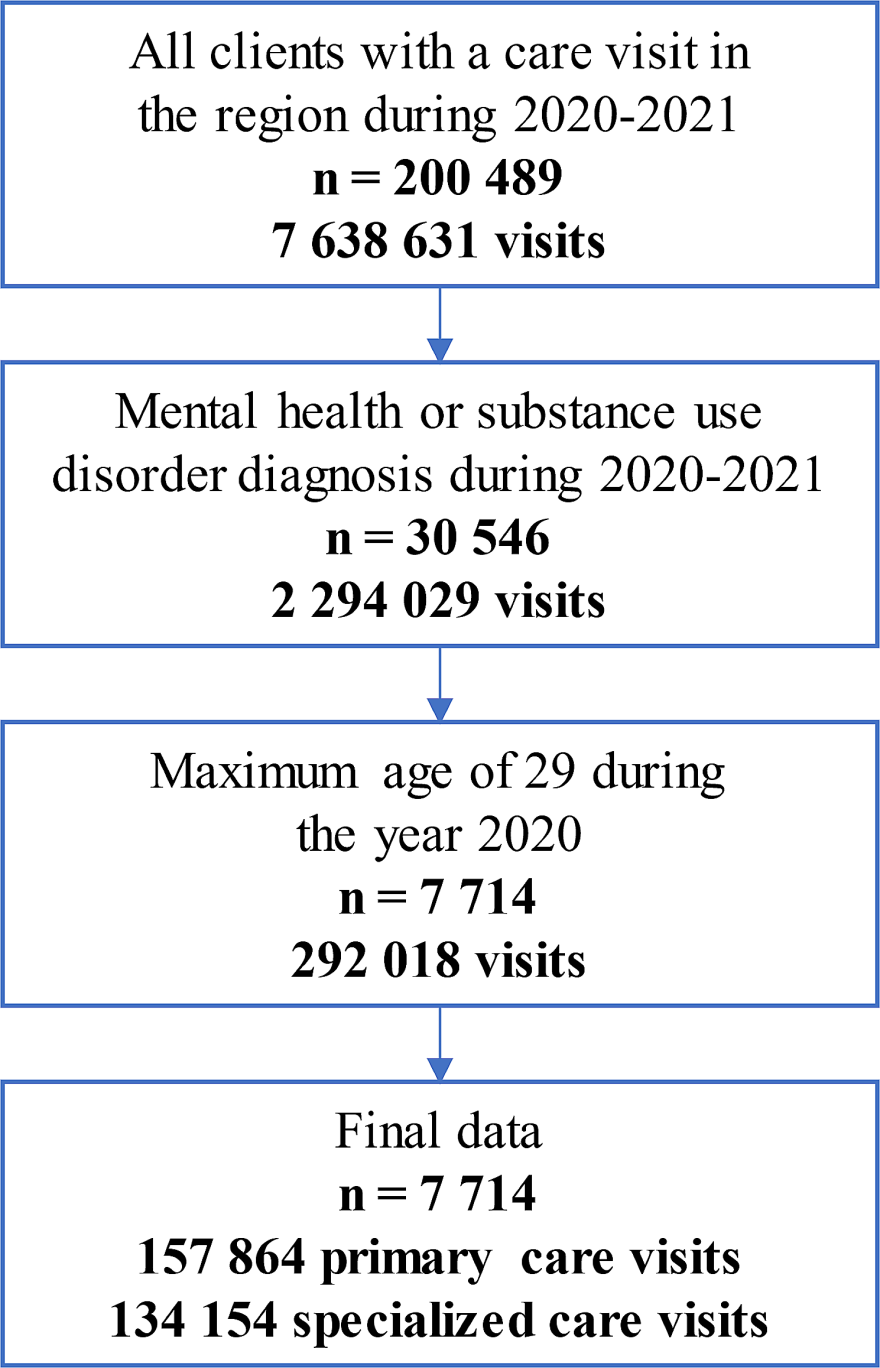
Data flowchart

The data were obtained from the Finnish national care registers. PHC visits were retrieved from the register of primary health care visits, and specialized care visits were retrieved from the care register for health care. The registries included all care visits in PHC and specialized care in the region. The service type categorization existing in the register was used, which differentiates PHC use mainly by the provider of the care, for example primary mental health services provided by mental health teams. The data were collected over two years, from January 2020 to December 2021. The care visits were limited to young adults, who were defined those aged less than 30 years in year 2020, in accordance with the Finnish Youth Act [29], which concerns those aged 7 to 29 years old. Only people who were diagnosed with a psychiatric or substance use disorder during the 2-year period were included (Table 1). To exclude services outside of scope, the service type “home care visits” was removed from the data. Additionally, to reduce the impact of COVID-19-related care, service types related to tests and vaccinations were excluded. The final dataset included 7 714 individuals with 157 864 primary care visits and 134 154 specialized care visits (Fig. 1).

### Measures

#### Variables used for profiling

Ten variables, each consisting of the number of visits to a specific service type or care specialization, were used for the cluster analysis. First, the service type of the visit to outpatient PHC was grouped into five distinct variables: (1) Ambulatory care (general consultations, including occupational healthcare and dental care), (2) mental health services, (3) substance use services, (4) student health services, and (5) other services, which included visits to child/family health clinics, health-related social work, and nonpsychiatric therapeutic services.

Second, for inpatient specialized care visits and hospitalizations taking place in a hospital setting, the level of the specialization of the care given (often the same as the physician’s specialization) was grouped into three categories: (1) general medicine, including internal and acute medicine, (2) psychiatry, including substance use, and (3) other, which included surgery, neurology, and physiatry. Third, care visits in acute care services were used, separately for PHC (consultation without appointment in care centers during office hours) and specialized care (around-the-clock emergency departments).

#### Patient and care-related characteristics

The explanatory variables in the statistical analysis were (1) individual characteristics, including age, sex, the municipality of residence, and the specific diagnosis received, and (2) care related characteristics, including the professional’s occupation and continuity of care.

Age and sex were determined from the registry data. No information on the municipality of residence was available; therefore, the municipality with the most ambulatory care visits in PHC was assumed to be the municipality of residence. The municipalities were grouped based on their population size: (1) largest municipality (58%), (2) over 10 000 people (3 municipalities, 28%), and (3) under 10 000 people (6 municipalities, 14%). The diagnoses related to mental health and substance use (Table 1) were grouped into eight groups, as described by Hakulinen and colleagues [30]. Due to the challenge of defining the principal disorder, all diagnoses in the register during the two-year period were accounted for.

Visits to different professionals (physician, nurse, other), irrespective of service type, visit type (remote visits and on-site visits), and care specialization, were included for both PHC and specialized care. The physicians included both general practitioners and specialists. Nurses included registered nurses and nurse specialists, such as psychiatric nurses. The category “other” included psychologists, social workers, psychotherapists, and other therapists.

Continuity of care was calculated separately for PHC and for specialized care by subtracting the number of unique professional IDs among all visits from the number of total visits divided by the number of total visits. For example, a value of 0.50 means that half of the client’s visits were given by repeated professionals, and a value of 0 means that all visits were given by different professionals.

#### Statistical analyses

First, to detect the care use profiles, exploratory cluster analysis was conducted using the K-Means clustering method, which classifies participants into groups minimizing intra-class variation and maximizing inter-class variation [31]. K-Means clustering was chosen as a method in accordance with previous research that had similar research aims and utilized register-based data [25, 26]. The number of clusters was determined using both the elbow method (minimizing intra-class variation per added cluster) and the average silhouette approach (optimizing the placement of objects within the cluster).

Second, to assign names for the clusters, descriptive statistics of background variables and mean service utilization were compared. Finally, we used multinomial logistic regression to analyze the associations between different individual-level variables and membership in different clusters. All assumptions were met. As the data consists of routinely collected care documentation, missingness and incompleteness in the information of the care visits is not unusual. In our original data approximately 2.5% of the values (in total 4019) in the service type (PHC) were missing, and in the other variables missingness was marginal. Care visits with relevant missing data were omitted from the analyses. In addition, outliers in care visits were examined and deemed valid. R version 4.2.2 for Windows [32] with packages dplyr [33] and nnet [34] were used for the data manipulation and analysis.

**Table 1:** Psychiatric diagnoses. In case an individual has multiple diagnoses, all are included

Psychiatric diagnoses	ICD-10 code	ICPC-2 code	*n* (%)
Substance use disorders	F10-F19	P15-P17, P19	797 (10.3)
Psychotic disorders	F20-F29	P72	358 (4.6)
Mood (affective) disorders	F30-F39	P73	3066 (39.7)
Anxiety disorders	F40-F48	P02, P74-P75, P78-P79, P82	4123 (53.4)
Eating disorders	F50	P86	246 (3.2)
Personality disorders	F60-F69		572 (7.4)
Developmental disorders	F80		282 (3.7)
Childhood onset disorders	F90-F98	P10-P13, P22-P23, P81	1168 (15.1)

## Results

The participants were 23 years old on average (SD 4) and a majority of them were women (63%) (Table 2). Most were residents in the region’s largest city (75%), with the rest residing either in municipalities with a population of over 10 000 (18%) or in municipalities with a population under 10 000 (7%). The most common psychiatric diagnoses were anxiety and mood disorders (53% and 40%, respectively). Fifteen and ten% of the participants were diagnosed with childhood onset and substance use disorders, respectively. Other diagnoses were relatively uncommon (7% or less).

The participants had on average 21 PHC visits (SD 22) and 17 specialized care visits (SD 29) during the two-year time period, with a range from 0 to 551 for PHC and from 0 to 544 for specialized care. The most common PHC service type was ambulatory (mean 11, SD 14), followed by psychiatric (mean 3, SD 6) and other (mean 2, SD 6). For hospital visits, psychiatric and general care specializations were most common (mean 11, SD 25 and mean 5, SD 7). Acute care and emergency department use was low (mean 1, SD 3 and mean 2, SD 3).

**Table 2:** Participant characteristics (*n* = 7714)

	Young adults diagnosed with a mental and/or substance use disorder *n* = 7714		
	Mean / %	SD	Min – max
**Age (at year 2020)**	23.1	3.8	16–29
**Female (%)**	62.9		
**Municipality of residence (%)**			
Largest	75.4		
> 10k population	17.8		
< 10k population	6.8		
**Psychiatric diagnoses (%)**			
Substance use disorders	10.3		
Psychotic disorders	4.6		
Mood disorders	39.7		
Anxiety disorders	53.4		
Eating disorders	3.2		
Personality disorders	7.4		
Developmental disorders	3.7		
Childhood onset disorders	15.1		
**Primary healthcare visits (years 2020–2021)**			
Ambulatory	11.4	13.7	0–449
Psychiatric	2.7	5.7	0–74
Substances	0.9	4.7	0–127
Student	1.7	4.2	0–117
Other	2.0	5.5	0–71
Acute care	1.1	3.0	0–90
Total	20.5	22.4	0–551
**Specialized care visits (years 2020–2021)**			
General	4.9	7.2	0–138
Psychiatric	10.6	25.4	0–529
Other	1.1	4.0	0–62
Emergency department	1.5	3.3	0–100
Total	17.4	28.7	0–544
**Total visits in years 2020–2021**	37.9	40.1	1–647

### Cluster analysis

**Fig. 2:**
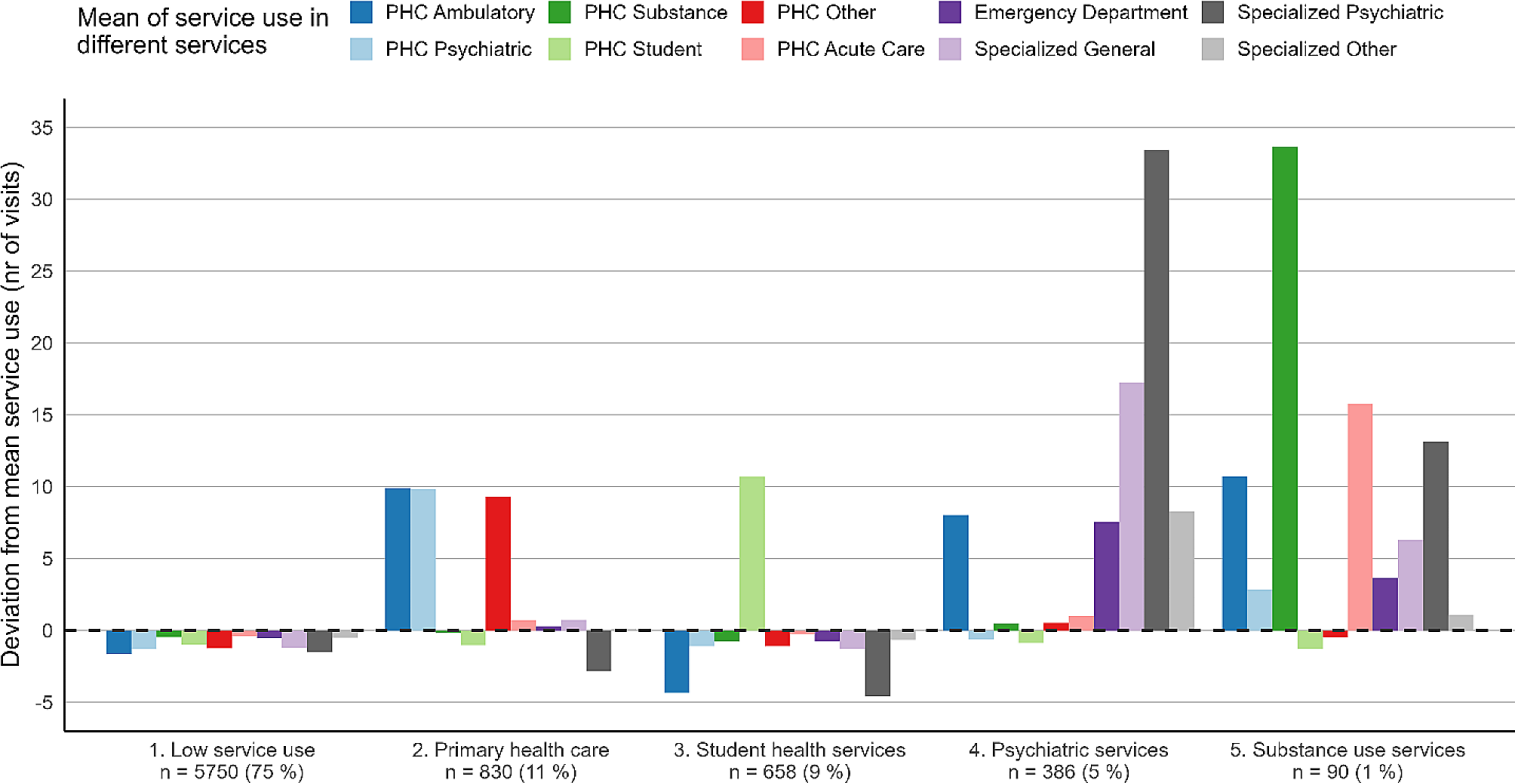
Identified profiles and their care utilization (*n* = 7714)

Five profiles were identified (Fig. 2). The largest profile group was named “Low service use” (*n* = 5750, 75%). The service utilization in this profile was on average low and consisted mainly of PHC ambulatory and psychiatric service visits. Majority of the participants (51%) in this profile had less than 11 visits in PHC during the study period, while 90% had less than 30 PHC visits (range: 0–259).

The second profile was named “Primary health care” (*n* = 830, 11%), because the participants belonging to this profile were characterized by high PHC use (mainly psychiatric services). In contrast, their use of specialized care services was low.

The third profile, named “Student health services”, included 658 persons (9% of the study sample). Among participants in this profile, service use was generally low, but the use of student care services was notably high.

The fourth profile was named “Psychiatric services” (*n* = 386, 5%). Participants belonging to this profile had used mainly psychiatric and general specialized care services in addition to primary care services. Additionally, participants belonging to this profile had a high number of emergency department visits.

The fifth and smallest profile group was named “Substance use services” (*n* = 90, 1%) because the service utilization of participants in this group was focused heavily on substance use (PHC) and psychiatric services in specialized care. The clients in this profile had substantial acute care service (PHC) and emergency department utilization.

### Multinomial logistic regression

The associations of age, sex, area of residence, psychiatric diagnosis, visits to different professionals, and continuity of care with profile membership were examined using multinomial logistic regression, with the largest profile group (Low service use) serving as the reference group (Table 3). In other words, the results below are in relation to the low service use profile.

**Table 3:** Multinomial logistic regression results, indicating associations with the identified profiles, as the largest profile (low service use) as the reference group

Reference: Profile 1	Profile 2	Profile 3	Profile 4	Profile 5
Low service use	Primary health care	Student health services	Psychiatric services	Substance use services
*n* = 5750	*n* = 830	*n* = 658	*n* = 386	*n* = 90
**Variable**	**OR (95% CI)**	**OR (95% CI)**	**OR (95% CI)**	**OR (95% CI)**
Age	1.04** **(1.01–1.07)**	0.82*** **(0.79–0.84)**	0.98 (0.95–1.02)	1.11* **(1.01–1.22)**
Male (ref.)				
Female	2.58*** (1.96–3.39)	1.99*** **(1.60–2.47)**	1.37* **(1.02–1.84)**	0.97 (0.46–2.04)
**Municipality of residence**:				
Largest (ref.)				
Population > 10k	0.51*** (0.38–0.67)	0.35*** **(0.27–0.46)**	0.48*** **(0.34–0.68)**	0.20*** **(0.08–0.48)**
Population < 10k	0.57** **(0.37–0.86)**	0.08*** **(0.04–0.15)**	0.60 (0.35–1.03)	0.09* **(0.01–0.82)**
**Diagnoses**:				
1 Substance use disorders	0.37*** (0.24–0.56)	0.28*** **(0.18–0.44)**	2.51*** **(1.80–3.50)**	58.91*** (15.79–219.78)
2 Psychotic disorders	0.83 (0.45–1.54)	0.37* **(0.17–0.83)**	1.63* **(1.04–2.54)**	1.55 (0.55–4.41)
3 Mood disorders	0.93 (0.74–1.17)	0.71** **(0.58–0.87)**	0.82 (0.62–1.07)	0.78 (0.37–1.64)
4 Anxiety disorders	1.01 (0.80–1.28)	0.84 (0.69–1.03)	0.92 (0.71–1.20)	0.66 (0.31–1.38)
5 Eating disorders	0.57 (0.27–1.21)	1.29 (0.77–2.16)	1.35 (0.82–2.22)	0.44 (0.03–6.22)
6 Personality disorders	0.80 (0.51–1.23)	1.16 (0.78–1.72)	1.17 (0.79–1.73)	1.84 (0.63–5.35)
8 Developmental disorders	0.60 (0.30–1.18)	0.62 (0.36–1.09)	0.54 (0.26–1.12)	0.13 (0.00–4.50)
9 Childhood onset disorders	0.67* **(0.49–0.93)**	1.00 (0.77–1.29)	0.88 (0.63–1.25)	1.45 (0.67–3.15)
**Continuity of Care index**:				
PHC	1.04*** **(1.03–1.05)**	1.05*** **(1.05–1.06)**	0.98*** **(0.97–0.98)**	1.02 (1.00–1.06)
Specialized care	1.00 (0.99–1.00)	0.99** **(0.99–1.00)**	1.01** **(1.00–1.01)**	0.99 (0.97–1.00)
**Professions (per visit)**:				
PHC physician	1.04*** **(1.02**–**1.06)**	1.01 (0.99–1.03)	1.09*** **(1.07–1.11)**	1.01 (0.96–1.06)
PHC nurse	1.29*** **(1.27**–**1.31)**	1.15*** **(1.13–1.17)**	1.13*** **(1.11–1.16)**	1.39*** **(1.35–1.44)**
PHC other	1.01 (1.00–1.02)	0.98** **(0.97–1.00)**	1.02*** **(1.01–1.03)**	0.87* **(0.76–0.98)**
Specialized care physician	1.04 (0.97–1.11)	0.94 (0.87–1.01)	1.38*** **(1.33–1.44)**	1.04 (0.88–1.21)
Specialized care nurse	0.98* **(0.97**–**1.00)**	0.99 (0.98–1.01)	1.01*** **(1.01–1.02)**	1.01 (1.00–1.02)
Specialized care other	0.99 (0.97–1.02)	1.02* **(1.00–1.03)**	1.02*** **(1.01–1.04)**	0.98 (0.90–1.07)

Bold = statistically significant (*p* < 0.05)

* = *p* < 0.05, ** = *p* < 0.01, *** = *p* < 0.001

Compared to the low service use profile, membership in the second profile (Primary health care) was statistically significantly associated with female sex (OR: 2.58 [95% CI 1.96–3.39]), older age (OR: 1.04 [95% CI 1.01–1.07]), and care visits to PHC nurses (OR: 1.29 [95% CI 1.27–1.31]) and physicians (OR: 1.04 [95% CI 1.02–1.06]).

The third profile (Student health services) membership was significantly associated with younger age (OR: 0.82 [95% CI 0.79–0.84]), female gender (OR: 1.99 [95% CI 1.60–2.47]), and a higher continuity of care index in PHC (OR: 1.05 [95% CI 1.05–1.06]). For diagnoses, three disorder diagnoses (substance use, psychotic, and mood disorders) were negatively associated with the profile membership.

Compared to the low service use profile membership, the members in the fourth profile group (Psychiatric services) were more likely to have substance use (OR: 2.51 [95% CI 1.80–3.50]) or psychotic (OR: 1.63 [95% CI 1.04–2.54]) disorders. Their service use was characterized especially by specialized care physician visits (OR: 1.38 [95% CI 1.33–1.44]), in addition to other increased care use.

The membership of the last profile, substance use services, was strongly associated with substance use disorder (OR: 58.91 [95% CI 15.79-219.78]) and nurse-focused service use in PHC (OR: 1.39 [95% CI 1.35–1.44]). In addition, the members in this profile were more likely to be of older age (OR: 1.11 [95% CI 1.01–1.22]), and while not a statistically significant association, the results point to potentially greater continuity of care in PHC (OR: 1.02 [95% CI 1.00-1.06]).

Finally, the municipality of residence was associated with profile membership. Compared to individuals living in the largest municipality, individuals living in smaller municipalities generally had significantly lower odds of being in all the other profiles compared to the individuals in the low care use profile.

## Discussion

In this study, our aim was to identify different service use profiles among young adults with a diagnosed psychiatric or substance use disorder, using register data of individual visits to different care services. Five distinct profiles were identified, with most of the participants belonging to the low service use profile. Health care use in this population seems to follow a common pattern where a small minority use most of the services. For example, in a recent study by Nnoaham and Cann, over three quarters of the study population were included in the segments with low primary and specialized care utilization [25]. In another study, over half of the individuals in early intervention psychosis services had low care utilization in the consequent years [35]. In the present study, over half of the individuals in the low service use profile, and over third of the overall study participants, had less than six PHC visits per year, signifying that very low service use is rather common, even in this population. However, this could also include hard to reach clients disengaging from care, as low care utilization individuals have been found to eventually have higher than anticipated primary care utilization [35], reinforcing the significance of outreach, follow-up, and service access in this profile.

The service use of the members in the primary health care and student health services profiles focused on PHC with low specialized care utilization. As outlined in the Finnish mental health strategy [36], primary care services are designed as the initial service point for mental health and substance use clients, with referrals to specialized care when needed. The consequent care is coordinated between primary and specialized care, which can be seen in the last two profiles, psychiatric services and substance use services, where care utilization was high in both primary and specialized care. Similar trends have been noticed in the USA, with mental health care moving from specialists to general medical providers (primary health care) [37]. The results showcase the care pathway from low service use to PHC services, and finally if required, specialized care. This highlights the need for collaboration and integration between different levels of care, to successfully guide the clients through the available services and to fulfill their care needs in a timely fashion. In addition, care of the clients in the student health services needs to be continued seamlessly once they move to other care services after finishing their studies.

The service use in the members of the profiles may indicate that people diagnosed with a psychiatric or a substance use disorder have manifold care needs, as psychiatric care services were not the only care service used. Previous research has shown that people with a mental health and substance use disorder have an increased risk of physical health problems [38, 39]. In addition, the comorbidity of mental health and substance use diagnoses is high, and they vary by age, sex, and socioeconomic status [40]. To ensure that all care needs are met, care integration and seamless interdisciplinary collaboration between different care services and teams are needed, as according to the results, the care received especially by the profiles with higher care utilization was fragmented into multiple different services and service categories. Comparable results have been found in previous segmentation studies, with a small proportion of the population utilizing many services both in quality and quantity [25, 26], underlining the importance of information transfer among different levels of care, and continuous treatment and care planning.

Female gender was significantly associated with membership in the primary health care and student health services profiles, a result in line with previously found profiles [37, 41, 42]. Findings from Finland show that the proportion of individuals with generalized anxiety is significantly higher among young women [43], and in addition, psychological stress has been reported to be greater among women than men in the adult population [44]. While internalizing mental disorders are more common among women [45], other relevant factors could potentially be involved. Previously, a significant gender gap has been identified in help-seeking behavior [46]. Potential barriers to men’s help-seeking and service use might include avoidance behavior, feelings of embarrassment and anxiety, and communication problems with healthcare personnel [47]. However, as noted by Pattyn and colleagues [48], structured social norms, upheld by both men and women, also contribute to the gender gap in service use. Measures are required to meet the care needs of men with mental health and substance use disorders, possibly through the use of low-threshold services and helping raise awareness of serious symptoms.

Living in smaller municipalities was negatively associated with belonging to other than the low care utilization profiles, which is in line with previous research linking urban living with an increased prevalence of psychiatric disorders [49, 50]. In the present study, the results might also be explained by young adults, especially students, preferring to live in urban areas, or centralized services affecting service use, indicating the importance of adequate access to services, irrespective of place of residence.

As may be expected, participants with a diagnosed substance use disorder overwhelmingly belonged to the substance use services profile. Membership in the profile was additionally associated with older age, corroborating existing research indicating a considerable gap in the onset of substance use disorder and treatment seeking [51]. The size of the profile group was very small, and while substance use disorders are increasingly common [52], the case might be that a significant portion of users were not diagnosed or in treatment or were classified into other profiles. Indeed, having a diagnosed substance use disorder also increased the odds of belonging to the psychiatric services profile. In addition, it is also known that some people who have a substance use disorder are treated primarily in social services, which we had no data on. Regardless, actions that help substance users enter treatment earlier, such as low-threshold services and community outreach, are needed to reduce the potential adverse social, psychological, and health related outcomes associated with substance use among young people [53].

Membership in the student health services profile was associated with higher continuity of care in primary health care. This might be explained by the small number of potential service contacts in each school or student health services center, resulting in care visits often being made to the same professional. In addition, better continuity of care in PHC was associated with higher odds of being a member of the primary health care profile and possibly in the substance use services profile. Members in these profiles might be directed to smaller mental health and/or substance use teams when needing care, which could explain the greater proportion of repeated professionals in their visits. While no clear associations have been established between continuity of care and clinical outcomes for people with mental health and substance use disorders [54], individual studies have shown the potential of continuity of care to strengthen therapeutic relationships [55, 56] and possibly reduce specialized care costs [57]. As such, establishing multidisciplinary care teams focused solely on mental and/or substance use disorders might at minimum improve the patient-to-professional contacts.

This study provides important insights into the care utilization patterns of young adult clients with mental health or substance use disorders. The heterogeneous and complex nature of service utilization in this population has implications for service delivery and care integration. Future research should aim to include socioeconomic factors, which likely affect the utilization of mental health and substance use services. In addition, members of the profiles with potential unmet care needs need urgent attention. To reduce unmet needs, targeted interventions and approaches are needed to raise awareness of mental health and substance use symptoms and available care, especially for young men and for those with substance use disorders.

### Strengths and limitations

The strengths of the study include the use of a large dataset containing all registered visits to both primary and specialized care from two years. The data included robust information on the service type, specialization, and occupation of the professional at each visit. The quality of the Finnish care registries (previously hospital discharge register) has been found to be satisfactory to very good [58, 59]. The visit numbers and diagnoses in the sample were comparable to the national averages [24, 60], and the region examined is fairly similar to most other regions suggesting adequate generalizability.

While the dataset used was comprehensive, it had some limitations. Data from student mental health services and some private sector health service providers were incomplete in years 2020–2021, potentially resulting in the misclassification of some participants. The exclusion of social services and the third sector could affect the proportions of each profile and potentially underestimate the overall service use, especially in chronic and substance use disorders. The data were from years 2020 and 2021, which were the height of the COVID-19 pandemic in Finland. The demand for psychiatric care services increased during the pandemic [61], potentially leading to diagnoses attributable to it. However, the provision of care was not significantly affected [61], indicating that the care use remained stable. In addition, to limit the effect of the pandemic on the results, care visits related to vaccinations and testing were excluded.

Next, as the inclusion criterion was a diagnosis in the years 2020–2021, some patients might have been diagnosed earlier, and if the diagnosis was not repeated in subsequent visits, they might have been excluded. Individuals diagnosed late in the two-year period might have unusually low service utilization, as they have not yet entered treatment, which may somewhat inflate the proportion of the low service use profile. In addition, as no principal diagnosis was defined, all diagnoses found in the data were treated equally. Importantly, the care registers include only the occurred visits, and consequently, unmet needs could not be measured. The continuity of care index used was crude and one-dimensional, and thus might not entirely capture the construct as a multidimensional concept [62]. Lastly, socioeconomic background data were not included. Incorporating these factors in future studies could help further analyze the determinants of care utilization.

## Conclusions

The analysis of the care utilization of young adults diagnosed with a psychiatric or substance use disorder resulted in the identification of five remarkably different profiles. Majority of the participants had low care utilization, potentially indicating unmet care needs. The profiles varied significantly based on individual characteristics and care utilization, suggesting that tailored care solutions are important. The complex nature of service utilization found has implications for service delivery. For example, increasing the availability of low-threshold services could help increase access to mental health and substance use services in primary care, especially among young men. The profiles utilizing the most services highlight the significance of patient-oriented integrated services for this vulnerable population.

## Data Availability

The data used in the current study may be obtained from the Finnish Institute for Health and Welfare. Restrictions apply to the availability of these data, which were used under license for this study. For information on accessing the data, see www.thl.fi.

## Abbreviations

PHC: Primary health care
NGO: Non-governmental organization
ICD: International classification of diseases
ICPC: International classification of primary care
SD: Standard deviation
OR: Odds ratio
CI: Confidence interval

## References

[1] Weems CF, Silverman WK, Beauchaine TP, Hinshaw SP (2013). Anxiety disorders. Child and adolescent psychopathology.

[2] World Health Organization. World mental health report: Transforming mental health for all [Internet]. World Health Organization. 2022. https://www.who.int/publications/i/item/9789240049338.

[3] Kauhanen L, Wan Mohd Yunus WMA, Lempinen L, Peltonen K, Gyllenberg D, Mishina K (2023). A systematic review of the mental health changes of children and young people before and during the COVID-19 pandemic. Eur Child Adolesc Psychiatry.

[4] Kuitunen I, Uimonen MM, Ponkilainen VT, Mattila VM (2023). Primary care visits due to mental health problems and use of psychotropic medication during the COVID-19 pandemic in Finnish adolescents and young adults. Child Adolesc Psychiatry Ment Health.

[5] Clayton S (2020). Climate anxiety: psychological responses to climate change. J Anxiety Disord.

[6] Limone P, Toto GA, Messina G (2022). Impact of the COVID-19 pandemic and the Russia-Ukraine war on stress and anxiety in students: a systematic review. Front Psychiatry.

[7] Narusyte J, Ropponen A, Wang M, Svedberg P (2022). Sickness absence among young employees in private and public sectors with a history of depression and anxiety. Sci Rep.

[8] Knapp M (2000). Schizophrenia costs and treatment cost-effectiveness. Acta Psychiatr Scand.

[9] Leibson CL (2001). Use and costs of Medical Care for children and adolescents with and without Attention-Deficit/Hyperactivity disorder. JAMA.

[10] Palmu R, Partonen T (2023). Severe or chronic disease in childhood predicts suicidality and links to anxiety in young adulthood. Nord J Psychiatry.

[11] Carpenter JS, Iorfino F, Cross S, Nichles A, Zmicerevska N, Crouse JJ (2020). Cohort profile: the brain and mind centre optymise cohort: tracking multidimensional outcomes in young people presenting for mental healthcare. BMJ Open.

[12] Iorfino F, Scott EM, Carpenter JS, Cross SP, Hermens DF, Killedar M (2019). Clinical stage transitions in persons aged 12 to 25 years presenting to Early Intervention Mental Health Services with Anxiety, Mood, and psychotic disorders. JAMA Psychiatry.

[13] Honkanen O, Sirniö O, Vaalavuo M. Mielenterveyden häiriö nuoruudessa on yhteydessä suurempiin terveyspalvelujen käytöstä aiheutuviin kustannuksiin aikuisena [Mental disorder in adolescence is associated with higher levels of healthcare costs in adulthood]. THL Tutkimuksesta tiiviisti [Internet]. 2023;38. https://urn.fi/URN:ISBN:978-952-408-126-9.

[14] United Nations Children’s Fund. The State of the World’s Children 2021: On My Mind Promoting, Protecting and Caring for Children’s Mental Health [Internet]. United Nations; 2021 [cited 2024 Jan 9]. (State of the World’s Children). https://www.un-ilibrary.org/content/books/9789210010580.

[15] World Health Organization. Comprehensive mental health service networks: promoting person-centred and rights-based approaches [Internet]. World Health Organization. 2021. https://apps.who.int/iris/handle/10665/341646.

[16] OECD. A New Benchmark for Mental Health Systems: Tackling the Social and Economic Costs of Mental Ill-Health [Internet]. OECD. 2021 [cited 2023 Nov 7]. (OECD Health Policy Studies). https://www.oecd-ilibrary.org/social-issues-migration-health/a-new-benchmark-for-mental-health-systems_4ed890f6-en.

[17] McGorry PD, Mei C, Chanen A, Hodges C, Alvarez-Jimenez M, Killackey E (2022). Designing and scaling up integrated youth mental health care. World Psychiatry.

[18] Menear M, Girard A, Dugas M, Gervais M, Gilbert M, Gagnon MP. Personalized care planning and shared decision making in collaborative care programs for depression and anxiety disorders: A systematic review. Hutchinson G, editor. PLoS ONE. 2022;17(6):e0268649. 10.1371/journal.pone.0268649 PMC9187074 35687610

[19] Tynkkynen LK, Keskimäki I, Karanikolos M, Litvinova Y, Finland. Health system summary, 2023 [Internet]. Copenhagen: World Health Organization. Regional Office for Europe; 2023. (Health system summary;). https://iris.who.int/handle/10665/366710.

[20] Linnaranta O, Ranta K, Marttunen M, Aalto-Setälä T, Ståhle M, Suvisaari J et al. A national implementation of interpersonal counselling, adolescent version (IPC-A) in Finland. In: Psychiatria Fennica. 2022. pp. 24–53.

[21] Ministry of Social Affairs and Health. National Mental Health Strategy and Programme for Suicide Prevention 2020–2030. Publications of the Ministry of Social Affairs and Health [Internet]. 2020;15. http://urn.fi/URN:ISBN:978-952-00-5401-4.

[22] Arajärvi M, Mönkkönen K, Kekoni T, Toikko T. Psychosocial social work as part of interdisciplinary collaboration and care need assessment in psychiatric outpatient care. Nordic Social Work Res. 2023;1–18.

[23] Huhta H, Tourunen J, Kaskela T, Takala J, Helfer A, Jurvanen S et al. Expanding the understanding of low-threshold services for young people [Internet]. Prime Minister’s Office; 2023. (Government’s analysis, assessment and research activities). Report No.: 2023:9. julkaisut.valtioneuvosto.fi/bitstream/handle/10024/164402/9-2023-Expanding%20the%20understanding%20of%20low-threshold%20services%20for%20young%20people.pdf.

[24] Forsell M. Lasten ja nuorten mielenterveysperusteinen tutkimus ja hoito 2020: Lähes joka viidennellä 18–22-vuotiaalla oli mielenterveyteen liittyvä käynti julkisessa terveydenhuollossa vuonna 2020 [Mental health research and care for children and young people 2020: Nearly one in five 18–22 year olds had a mental health-related care visit in 2020]. THL Tilastoraportti [Internet]. 2022;21. https://urn.fi/URN:NBN:fi-fe2022060342738.

[25] Nnoaham KE, Cann KF (2020). Can cluster analyses of linked healthcare data identify unique population segments in a general practice-registered population?. BMC Public Health.

[26] Vuik SI, Mayer EK, Darzi A (2016). Patient segmentation analysis offers significant benefits for Integrated Care and Support. Health Aff.

[27] Tiirinki H, Sulander J, Sinervo T, Halme S, Keskimäki I (2022). Integrating Health and Social Services in Finland: Regional approaches and Governance models. Int J Integr Care.

[28] Holster T, Nguyen L, Häkkinen U. The role of occupational health care in ambulatory health care in Finland. Nordic J Health Eco [Internet]. 2022 Jul 23 [cited 2023 May 9]; https://journals.uio.no/NJHE/article/view/8561.

[29] Nuorisolaki [Youth Act] [Internet]. 1285/2016. (Finland). https://www.finlex.fi/fi/laki/alkup/2016/20161285.

[30] Hakulinen C, Elovainio M, Arffman M, Lumme S, Pirkola S, Keskimäki I (2019). Mental disorders and long-term labour market outcomes: nationwide cohort study of 2 055 720 individuals. Acta Psychiatr Scand.

[31] Hartigan JA, Wong MA, Algorithm (1979). AS 136: a K-Means Clustering Algorithm. Appl Stat.

[32] R Core Team. A language and environment for statistical computing. R Foundation for Statistical Computing, Vienna, Austria [Internet]. 2020; https://www.R-project.org/.

[33] Wickham H, François R, Lionel H, Müller K, Vaughan D. dplyr: A Grammar of Data Manipulation. https://dplyr.tidyverse.org [Internet]. 2023; https://github.com/tidyverse/dplyr.

[34] Venables W, Ripley B. Modern Applied Statistics with S, Fourth edition [Internet]. Springer, New York; 2002. https://www.stats.ox.ac.uk/pub/MASS4/.

[35] O’Driscoll C, Shaikh M, Finamore C, Platt B, Pappa S, Saunders R (2021). Profiles and trajectories of mental health service utilisation during early intervention in psychosis. Schizophr Res.

[36] Linnaranta O, Strand T, Suvisaari J, Partonen T, Solin P. Mielenterveysstrategia 2020–2030: Toimeenpanon ensimmäiset vuodet ja yhteisen tekemisen tahto [Mental Health Strategy 2020–2023: The first years of implementation and the will to work together]. THL Työpaperi [Internet]. 2022;55. https://urn.fi/URN:ISBN:978-952-343-990-0.

[37] Wang PS, Demler O, Olfson M, Pincus HA, Wells KB, Kessler RC (2006). Changing Profiles of Service Sectors Used for Mental Health Care in the United States. AJP.

[38] Firth J, Siddiqi N, Koyanagi A, Siskind D, Rosenbaum S, Galletly C (2019). The Lancet Psychiatry Commission: a blueprint for protecting physical health in people with mental illness. Lancet Psychiatry.

[39] Pizzol D, Trott M, Butler L, Barnett Y, Ford T, Neufeld SA (2023). Relationship between severe mental illness and physical multimorbidity: a meta-analysis and call for action. BMJ Ment Health.

[40] Jacobi F, Höfler M, Siegert J, Mack S, Gerschler A, Scholl L (2014). Twelve-month prevalence, comorbidity and correlates of mental disorders in Germany: the Mental Health Module of the German Health Interview and examination survey for adults (DEGS1‐MH). Int J Methods Psych Res.

[41] Crable EL, Drainoni ML, Jones DK, Walley AY, Milton Hicks J (2022). Predicting longitudinal service use for individuals with substance use disorders: a latent profile analysis. J Subst Abuse Treat.

[42] Simo B, Bamvita JM, Caron J, Fleury MJ (2018). Patterns of Health Care Service utilization by individuals with Mental Health Problems: a predictive cluster analysis. Psychiatr Q.

[43] Kiviruusu O, Haravuori H, Lindgren M, Therman S, Marttunen M, Suvisaari J (2023). Generalized anxiety among Finnish youth from 2013 to 2021—Trend and the impact of COVID-19. J Affect Disord.

[44] Suvisaari J, Appelqvist-Schmidlechner K, Solin P, Parikka S, Koskela T, Ikonen J. Aikuisväestön mielenterveys ja avun hakeminen mielenterveysongelmiin – FinSote 2020 [Adult mental health and help-seeking for mental health problems - FinSote 2020]. THL Tutkimuksesta tiiviisti [Internet]. 2021;42. https://urn.fi/URN:ISBN:978-952-343-698-5.

[45] Boyd A, Van De Velde S, Vilagut G, De Graaf R, O׳Neill S, Florescu S (2015). Gender differences in mental disorders and suicidality in Europe: results from a large cross-sectional population-based study. J Affect Disord.

[46] Galdas PM, Cheater F, Marshall P (2005). Men and health help-seeking behaviour: literature review. J Adv Nurs.

[47] Yousaf O, Grunfeld EA, Hunter MS (2015). A systematic review of the factors associated with delays in medical and psychological help-seeking among men. Health Psychol Rev.

[48] Pattyn E, Verhaeghe M, Bracke P (2015). The gender gap in mental health service use. Soc Psychiatry Psychiatr Epidemiol.

[49] Xu J, Liu N, Polemiti E, Garcia-Mondragon L, Tang J, Liu X (2023). Effects of urban living environments on mental health in adults. Nat Med.

[50] Peen J, Schoevers RA, Beekman AT, Dekker J (2010). The current status of urban-rural differences in psychiatric disorders. Acta Psychiatr Scand.

[51] Blanco C, Iza M, Rodríguez-Fernández JM, Baca-García E, Wang S, Olfson M (2015). Probability and predictors of treatment-seeking for substance use disorders in the U.S. Drug Alcohol Depend.

[52] Degenhardt L, Glantz M, Evans-Lacko S, Sadikova E, Sampson N, Thornicroft G (2017). Estimating treatment coverage for people with substance use disorders: an analysis of data from the World Mental Health surveys. World Psychiatry.

[53] Hall WD, Patton G, Stockings E, Weier M, Lynskey M, Morley KI (2016). Why young people’s substance use matters for global health. Lancet Psychiatry.

[54] Puntis S, Rugkåsa J, Forrest A, Mitchell A, Burns T (2015). Associations between Continuity of Care and Patient outcomes in Mental Health Care: a systematic review. PS.

[55] Biringer E, Hartveit M, Sundfør B, Ruud T, Borg M (2017). Continuity of care as experienced by mental health service users - a qualitative study. BMC Health Serv Res.

[56] Sweeney A, Rose D, Clement S, Jichi F, Jones IR, Burns T (2012). Understanding service user-defined continuity of care and its relationship to health and social measures: a cross-sectional study. BMC Health Serv Res.

[57] Barker I, Steventon A, Deeny SR. Association between continuity of care in general practice and hospital admissions for ambulatory care sensitive conditions: cross sectional study of routinely collected, person level data. BMJ. 2017;j84. 10.1136/bmj.j84 28148478

[58] Haukka J. Finnish health and social welfare registers in epidemiological research. Nor J Epidemiol [Internet]. 2009 Oct 14 [cited 2023 Oct 12];14(1). https://www.ntnu.no/ojs/index.php/norepid/article/view/284.

[59] Sund R (2012). Quality of the Finnish Hospital Discharge Register: a systematic review. Scand J Public Health.

[60] Haula T, Laukkonen ML, Holster T, Korajoki M, Suvisaari J. Mielenterveys- ja päihdepalvelujen alueellisen käytön ja tarpeen arviointi [Assessment of the regional use and the need for mental health and substance use services]. THL Työpaperi [Internet]. 2023;24. https://urn.fi/URN:ISBN:978-952-408-121-4.

[61] Kestilä L, Kapiainen S, Mesiäislehto M, Rissanen P. Covid-19-epidemian vaikutukset hyvinvointiin, palvelujärjestelmään ja kansantalouteen - Asiantuntija-arvio, kevät 2022 [Impact of the Covid-19 epidemic on welfare, the service system, and the economy - Expert assessment, spring 2022]. THL Raportti [Internet]. 2022;4. https://urn.fi/URN:ISBN:978-952-343-865-1.

[62] Weaver N, Coffey M, Hewitt J (2017). Concepts, models and measurement of continuity of care in mental health services: a systematic appraisal of the literature. Psychiatric Ment Health Nurs.

